# Volatile Organic Compounds: A Promising Tool for Bed Bug Detection

**DOI:** 10.3390/ijerph20065214

**Published:** 2023-03-22

**Authors:** Mohammad Akhoundi, Dahlia Chebbah, Nohal Elissa, Sophie Brun, Julie Jan, Isabelle Lacaze, Arezki Izri

**Affiliations:** 1 Parasitology-Mycology Department, Avicenne Hospital, AP-HP, Sorbonne Paris Nord University, 93000 Bobigny, France; 2 Service Parisien de Santé Environnementale (SPSE), Sous-Direction de la Santé Environnementale et de la Prévention (SDSEP), Direction de la Santé Publique (DSP)—Mairie de Paris, 75019 Paris, France; 3 Agence Régionale de Santé (ARS) Île-de-France, 35, Rue de la Gare, CEDEX 19, 75935 Paris, France; 4 Centre Scientifique et Technique du Bâtiment (CSTB), Direction Santé Confort, Division Qualité Sanitaire des Ouvrages, 84, Avenue Jean Jaurès, CEDEX F-77447, 77420 Marne-la-Vallée, France; 5 Unité des Virus Émergents (UVE: Aix-Marseille Univ-IRD 190-Inserm 1207-IHU Méditerranée Infection), 13000 Marseille, France

**Keywords:** bed bugs, chemical ecology, semiochemicals, olfactory sensilla, VOC detection, pheromones

## Abstract

The recent decades’ resurgence of bed bugs as a public health concern in industrialized countries has driven an increased interest on new sustainable insecticide-free methods to monitor and control these ectoparasites. Current methods of detection rely mainly on visual inspection or canine scent detection, which are methods that are time-consuming, require experience, are non-specific or require costly mission repetitions. Volatile organic compounds (VOCs) are considered an environmentally friendly alternative and a promising approach for bed bug detection. An overview of the released literature on VOCs, their chemical characteristics and their role in bed bugs’ intra- and inter-species communications allowed us to highlight the identification of 49 VOCs in *Cimex lectularius* (23 molecules) and *C. hemipterus* (26), which are emitted by both sexes during diverse compartments including aggregation (46), mating (11), defense (4), etc., and all life stages including exuviae or dead bed bugs as a principal indicator of infestation. The latter has a great importance for application of these semiochemicals in successful detection and control management of bed bugs and to prevent their further dispersion. This approach has the advantage of more reliability compared to conventional detection methods with no need for repeated inspections, household furniture moving or resident rehousing for bed bugs’ VOC detection, which are commonly performed by active or passive sampling with absorbing tubes and analyzed by gas chromatography-based analytical platforms.

## 1. Introduction

Bed bugs, *Cimex lectularius* and *C. hemipterus* (Hemiptera: Cimicidae), are blood-sucking insects with a long history of presence in human communities. Since the late 1990s, there has been a resurgence of bed bugs worldwide particularly in industrialized countries in which increased international travel together with ineffective chemical insecticides play major roles in the spread of these ectoparasites [1,2,3]. Bed bugs are a major concern to public health and responsible for several clinical and psychological disorders. Bed bug bites can cause a wide spectrum of dermatological manifestations, varying from erythematous macules or papules to bullous eruptions [4]. Beside clinical issues, they are responsible for significant psychological disorders such as nightmares, anxiety, insomnia, paranoia and personal dysfunction [5]. In rare cases, bed bug bites in heavily infested dwellings can be considered a possible cause of chronic blood loss and anemia [6]. Furthermore, bed bug infestations pose severe challenges in elderly and low-income housing. They excrete large amounts of liquid feces into human dwellings which may alter the indoor microbial community composition [7]. These direct health impacts translate into potentially large social and economic costs [8,9]. Finally, they are responsible for multiple economic problems that affect cultural and tourism industries [10]. They are commonly found in beds, mattress, boxspring, walls cracks, crevices, electrical outlets or wooden furniture which can constitute microhabitats where bed bugs can hide to stay close to humans [2]. Bed bug infestations commonly occur in private or social dwellings, hospitals, hotels, touristic residences or public transportation [11]. Due to the nocturnal and discrete behavior of bed bugs, it is often difficult to find them through observation without additional aid particularly in the early stages of infestation.

Early detection of bed bugs is a prerequisite key factor in management of bed bug infestations and in reducing both the costs associated with bed bug management and the spread of bed bugs from infested dwellings to new locations [12]. Detection of low-level bed bug infestations is essential for early intervention, eradication and prevention of bed bugs spreading. Therefore, proper detection reduces management cost and time needed for control. Despite the importance of early detection, few effective tools and methods exist for detecting a low number of bed bugs [13,14]. Current methods of detection generally include (i) visual inspection, (ii) canine scent detection and (iii) monitoring methods/devices to monitor whether management should be continued or terminated.

Visual inspection is the most common but labor-intensive and time-consuming method, which requires experience and training. Due to the hidden behavior of bed bugs, visual inspection is not considered a reliable method, in particular in the case of low numbers of bed bug specimens in the initial steps of an infestation [12,15].

Canines have been used to detect pests including insects since the mid-1970s. The first use of dogs in the field was described to detect the odor of gypsy moth (Porthetria dispar) pheromones and eggs [16]. Afterward, canine detection of bed bugs has been considered an inspection tool which has been increasingly used in recent years. However, in a few investigations carried out on the sensitivity of this method to bed bug detection, the results were significantly controversial. Pfiester [17] stated a 95% sensitivity in detecting small numbers of bed bugs with no false indications using concealed bugs in hotel rooms, while Cooper et al. [18] reported a mean detection rate of 44% with a mean false-positive rate of 15%. The probability of a bed bug infestation being detected by trained canines was not associated with the level of bed bug infestation. Furthermore, it is a costly mission requiring repetition [18].

In the recent decades, monitoring methods/devices were the subject of several investigations, and multiple devices in combination with chemical or biochemical attractants have been developed for detecting bed bugs [19,20]. Thus, there has been increased interest in developing alternative or supplemental detection/monitoring methods. Currently, the need for more reliable and cost-effective detection methods is essential in bed bug infestation management [21,22].

Semiochemicals are the molecules produced by organisms including insects for intra- (pheromones) or inter-species (allomones, synomones or kairomones) communication. Volatile organic compounds (VOCs) are a group of semiochemicals in a gaseous phase with high vapor pressure. They include a large group of various chemical compounds emitted by living or non-living sources [23]. The resurgence of bed bug infestations and the inefficiency of chemical insecticides in many parts of the world have led to a renewed interest in VOCs as an alternative method of detection. The VOCs in bed bug were the subject of several investigations, mostly published in the last two decades [24,25,26]. Although they are helpful in enhancing our knowledge of the role and function of VOCs in bed bugs, some of them are restricted only to specific in vitro conditions or particular life stages without giving deep insight on the VOCs and their impact on bed bug compartments. These scattered pieces of information with occasionally controversial issues further complicate the puzzle. Moreover, the essential components of bed bugs’ VOCs need to be better characterized for use in the detection and management of bed bug infestations. Therefore, exhaustive characterization of VOCs released by bed bugs can be significantly helpful in developing accurate detecting methods and in the improvement of bed bug control practices. In this study, we overview the complete list of VOCs detected in bed bugs, the mechanism of their perception by bed bugs’ olfactory system and their impact on the bed bugs’ comportment. Moreover, we further concentrate on the various sampling and analytical methods of VOCs. Finally, we highlight the significance of VOCs as an ecofriendly alternative and their application as a promising approach in bed bug management.

### 1.1. Bed Bugs Chemical Ecology

Bed bug chemical ecology is a foremost concept in better understanding the biology of bed bugs and their communication with themselves, other insects and the environment and is critical to bed bug survival in a new environment. It deals with the chemical mechanisms that regulate both intra- and inter-specific interactions. It includes two major sections: (i) chemicals involved in the location of the host and (ii) interaction between bed bugs [27,28].

Bed bugs are able to find their host via multiple cues. Heat and CO_2_ are the most attractive cues used by bed bugs during host seeking [29,30], further confirmed by bed bug trapping studies [31,32,33]. Chemical cues associated with human bodies were the subject of several investigations in which over 400 compounds were identified as released by human skin [34,35]. Among these compounds, only some specific VOCs elicit a response from hematophagous insects [36]. Several aldehydes and 6-methyl-5-hepten-2-one were the only chemicals identified from human skin inducing an electrophysiological response from bed bugs [37]. This less sensibility can be explained in part by fewer odor-binding proteins and odorant receptors of bed bugs rather than other blood-feeding insects such as kissing bugs (*Rhodnius prolixus*) and mosquitoes (*Anopheles gambiae*, *Aedes aegypti*), correlating well with the low number of olfactory sensilla (44) present on bed bug antennae [22]. However, the interactions among bed bugs are mediated by the release of different chemical cues which result in diverse vital compartments (e.g., aggregation, alarm, etc.) in the life cycle of bed bugs [38,39].

### 1.2. Volatile Organic Compounds (VOCs)

VOCs are generally lipophilic molecules derived from primary or secondary metabolic process and classified according to molecular structure or functional group. These airborne metabolites commonly include diverse chemical groups such as aliphatic hydrocarbons, aromatic compounds, alcohols, ethers, esters, aldehydes, alkenes, ketones or terpenes [40]. They are composed of at least carbon and hydrogen. With high vapor pressure at room temperature (≥0.01 kPa), they are predominantly present in gaseous form. They have significant diffusion power given their volatility at standard temperature and pressure [41,42,43,44]. Since they are often emitted from living organisms, they include a number of carbons ranging from C2 to C20 with molecular weight less than 300 Daltons [45]. Other than VOCs from living origin (biogenic), there are some other compounds that do not occur naturally but are instead man-made, formed during industrial processes or combustion [46]. Furthermore, some VOCs are taxon-specific, whereas other VOCs appear to be common to many different bug families.

VOCs are released by many insects including bed bugs for intra- and inter-specific communication. They significantly influence reproduction, oviposition, prey location, defense behavior, aggregation and social organization [47,48]. The production of the mentioned molecules, depending on their target, varies widely across different insect orders. Some VOCs are species-specific while some others are general and secreted by other insect species as well. In bed bugs, VOCs are not only emitted by adult specimens but also by immature developmental stages. Furthermore, VOCs are emitted from exuviae or dead specimens. In an investigation on the VOCs in freshly shed exuviae of nymphal bed bugs (*C. lectularius*), four volatile aldehydes including (E)-2-hexenal, 4-oxo-(E)-2-hexenal, (E)-2-octenal and 4-oxo-(E)-2-octenal were detected [49]. They were secreted from dorsal abdominal glands with pocket-like structures on the exuviae. Therefore, the presence and accumulation of bed bug exuviae and their volatized aldehydes might mediate bed bugs’ interaction with their microhabitats and serve as an indicator of bed bug infestation [49].

In insects, the sensilla (sensory hairs) allow perception of signals present in the environment. They cover the surface of olfactory organs and carry the olfactory sensory neurons (OSNs). They are mainly located on the antennae and mouthparts but can be found throughout the body [50,51]. The VOCs present in the environment go into sensilla through cuticular pores and dissolve into sensillum lymph with embedded OSNs [52]. The surface of the OSNs include small (10–20 kDa), globular, extracellular target-binding proteins namely odorant binding proteins (OBP) that interact with penetrated VOCs inducing neuronal activity via fluctuations in the basal firing rate of the OSNs. Therefore, the OBPs play an important role as VOC transporters, solubilizing VOCs and pheromones from the surrounding air into the aqueous phase of the odor sensory organ. They transport VOCs through the sensillum lymph to olfactory receptor neurons (ORNs) which are coated with olfactory receptor (OR) proteins, located in dendrites of olfactory neurons. These OR proteins can bind to specific VOCs, leading to signal an olfactory response [52]. Therefore, the ORNs transduce chemical signals into electrical signals, resulting in appropriate behavioral responses [53].

**Figure 1:**
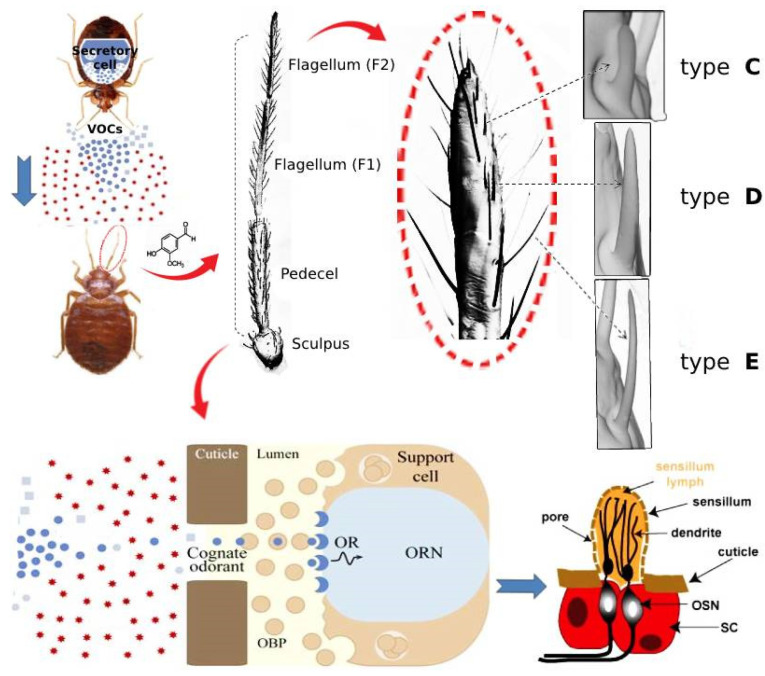
Schematic presentation of VOC perception in bed bugs.

The antennal sensilla of the bed bugs consist of three types of olfactory sensillum: type C (grooved peg sensilla), type D (smooth peg sensilla) and type E (hair-like sensilla) [54]. The type D sensillum is further characterized into Dα, Dβ and Dγ. Similarly, the type E sensillum is categorized into E1 and E2. Each type of the mentioned sensilla has a distinct response profile to a chemical panel [55]. Structural analysis of the terminal antennal segment of *C. lectularius* indicates a low number of olfactory sensilla (44 olfactory sensilla per antenna) [56]. The olfactory sensilla pattern (number and positions of the sensilla) is relatively consistent in males and females of *C. lectularius* [54]. It includes nine type C, 29 type E and one pair of each of Dα, Dβ and Dγ sensilla [57]. A simplified view of the VOC binding event in bed bugs is shown in Figure 1. It has been designed based on the findings of articles released recently [58,59].

Sensilla in bed bugs are divided into three categories: olfactory, gustatory and mechano-sensory [55]. These sensory organs can also be equipped with thermal or water receptors [60]. Thermal receptors have been already identified on the antennal pedicel of bed bugs [39]. Olfactory sensilla of bed bugs can detect VOCs and their olfactory information. They are further analyzed in the bugs’ brain and finally result in appropriate behavior.

**Figure 2:**
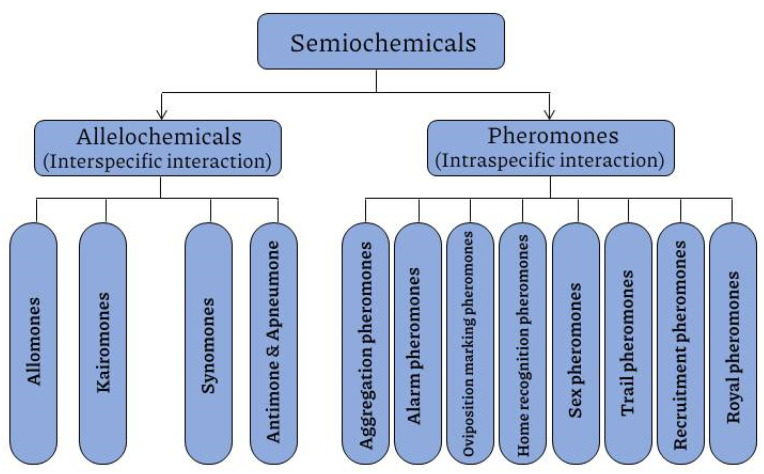
Diverse compounds of semiochemicals in hematophagous insects including bed bugs.

Among the biogenic VOCs in insects, most of them are secreted as pheromones (for intra-species communication) and some others as allelomones (inter-species communication). Pheromones are produced by an insect to perform a specific effect on another individual of the same species (intraspecific interactions), while allelomones are the compounds mediating interspecific interactions, affecting more insect species other than the species producing them [52,61]. VOCs are promising and can be used singly or in combination with other control strategies for monitoring and controlling insect pests in medical and agricultural systems. Their persistence is affected by a variety of environmental factors, such as temperature and hygrometry [57]. They may also release in response to an environmental stimulus. Knowledge of the composition of these semiochemicals and the forms in which they are released into the environment is crucial in using them for various control applications. These pheromones include aggregation pheromones, alarm pheromones, oviposition-deterrent pheromones, home recognition pheromones, sex pheromones, trail pheromones, recruitment pheromones and royal pheromones (Figure 2) [23].

***Aggregation*** is one of the most important phenomena in bed bugs living in a micro-ecological habitat. It was first identified by Levinson and colleagues in 1974 [62]. Aggregation pheromones mediate the formation of aggregation by attracting and/or arresting all conspecifics to the point of pheromone emission [50]. It provides more efficient resource use, an increased ability to find mates, protection from natural enemies and/or alteration of the microclimate allowing for protection from environmental conditions [50,63]. Thanks to aggregation, the bed bugs avoid dehydration more effectively than those living alone [64,65]. This phenomenon probably plays a remarkable role in long-time survival of bed bugs (sometimes for several months) without taking any blood meal in unsuitable environments. There are also benefits for juveniles in which the nymphs reared in groups develop faster than nymphs reared in isolation [66]. Aggregation behavioral response depends on the density of bed bugs, number of sensilla on the antenna and olfactory receptor neurons [67,68]. The aggregation pheromone is composed of multiple components with the concentration of each chemical being critical to the induction of clustering. It is mediated by VOCs secreted during adult and immature developmental stages as well as by male and female specimens. Bed bugs sense aggregation pheromones both by olfaction and contact chemoreception, indicating that the pheromone blend is composed of volatile and non-volatile components [22]. Based on olfactometry bioassays, no sexual dimorphism has been reported in the neuronal responses to aggregation pheromones and consequently in behavioral responses of male and female bed bugs [24,65]. In one of primitive investigations on the aggregation pheromones in bed bugs, 14 compounds with >100 pg abundance were detected in gas chromatography–mass spectrometry analyses. Among them, 10 compounds (nonanal, decanal, (E)-2-hexenal, (E)-2-octenal, (2E,4E)-octadienal, benzaldehyde, (+)- and (-)-limonene, sulcatone and benzyl alcohol) were reported to be essential components of the *C. lectularius* airborne aggregation pheromone [24]. In another laboratory survey of the bed bugs’ aggregation pheromones, five volatile components (dimethyl disulfide, dimethyl trisulfide, (E)-2-hexenal, (E)-2-octenal and 2-hexanone) were detected [65]. (E)-2-hexenal and (E)-2-octenal were reported to be essential components of bed bugs’ aggregation pheromones. The latter was confirmed in further investigations carried out by Gries et al. [65], Dery et al. [69] and Olson et al. [70]. Additionally, the bed bugs’ feces were reported to contain a variety of compounds which serve as a component of their aggregation pheromones [71]. The fecal matters influence aggregation behavior in *C. hemipterus*. (E)-2-hexenoic acid, hexanoic acid, (E)-2-hexenal and hexanal were found to exhibit aggregation in various stages of bed bugs’ life cycle [72]. In other investigation on these compounds, it seems that only virgin females responded to the aggregation pheromone (blood-fed females are not responsive to the aggregation pheromone), prompting the development of the hypothesis that female bed bugs aggregate less often in order to avoid traumatic insemination by males [24].

***Defense or alarm pheromones*** are typically known for their beneficial role in allowing individuals to escape predation leading to rapid dispersal of insects away from a potential threat. In bed bugs, alarm pheromones were firstly reported by Schildknecht and colleagues in 1964 [73] and are commonly used by bed bugs as a chemical defense against predation [54,60,74]. Alarm pheromones cause increased activity and dispersal in nymphs and adults. The alarm pheromones of bed bugs have a specific smell secreted from the first thoracic segment which is easily recognized by the human nose during emission [75,76]. (E)-2-hexenal and (E)-2-octenal were reported as the most abundant alarm pheromones in adult bed bugs [76]. The nymphs have two additional juvenile-specific compounds in their alarm pheromone blend: 4-oxo-(E)-2-hexenal and 4-oxo-(E)-2-octenal [38,65]. Alarm pheromones were demonstrated to be secreted in high doses in which their threshold for eliciting alarm behavior greatly exceeds the physiological detection threshold [54]. In a state of distress or alarm, bed bugs expel the contents of their scent glands, which stimulate locomotion of conspecifics [77]. Another role of alarm pheromones is during mating interactions. Nymphs and males release alarm pheromones to prevent sexual interactions from other conspecifics [75]. In addition, by using a blend of alarm pheromone, males are able to signal their identities to other males, avoiding erroneous mating attempts [75]. Finally, the bed bugs’ alarm pheromones have anti-fungal properties. A study testing (E)-2-hexenal and (E)-2-octenal against an isolate of *Metarhizium anisopliae* (Hypocreales: Clavicipitaceae) resulted in a significant inhibition of conidial viability [78].

***Oviposition marking pheromones* (*OMPs*)** are deposited by many parasitic and phytophagous insects immediately following egg-laying. These pheromones are recognized by tarsal and mouthpart receptors of gravid females inspecting potential oviposition sites [43]. They cause a change in egg-laying behavior of their conspecifics so that subsequent eggs are not deposited in resources that have already been used [79]. This effect results in a reduced time spent on the marked and previously utilized resource, reduced probability of oviposition, reduced competition for limited host resources (human or animal) among broods of conspecific organisms and superparasitism inhibition [79,80]. Although the effect of this pheromone depends on the fitness gain of this signal pheromone and the receiver species, little is known about this pheromone in bed bugs.

***Home recognition pheromones*** are mostly common in social insect colonies. For instance, bee queens produce a scent-mark to enable workers to recognize the colony [81]. The role of this pheromone is largely unknown in the bed bugs. However, it has been demonstrated that female bed bugs secret marking pheromones that may help them to find their harborages [64].

***Mating* (*sexual*)** behavior in bed bugs is a traumatic manner for females, with males piercing their abdominal cavity directly [82]. On the other side, mating in bed bugs is closely associated with the completion of blood feeding. During feeding, female bed bugs become too engorged to protect their exposed abdomen from males. Therefore, bed bug mating is primarily based on vision, and males are attracted to engorged bugs [1,83]. In many mating systems, females have a lower optimal mating rate than males and will acquire adaptations to resist mating. In response, males acquire adaptations to overcome female mating resistance. Therefore, adult bed bugs release a pheromone to encourage/dissuade adult males from mating. Females are known to emit chemicals during male copulation attempts [84]. Experiments involving females with their scent glands have shown that exposure to a mixture of (E)-2-hexenal and (E)-2-octenal in a 2:5 ratio can deter males from mating with manipulated females, whereas ratios of 1:1 and 5:4 (male- and female-specific ratios) did not have the same effect [85]. Although copulation between an adult and a nymph is reproductively ineffective, bed bug nymphs (*C. lectularius*) produce a chemical signal that interrupts the attempts of adult males to mate with them [85].

***Trail pheromones*** are often a multipurpose chemical secretion that leads members of the same species toward a food source, while representing a territorial mark of an allomone to other insects outside of the species [86]. Trail pheromones are often incorporated with secretions of more than one exocrine gland to produce a higher degree of specificity [87]. Considered one of the primary chemical signaling methods in which many social insects depend on, trail pheromone deposition can be considered as one of the main facets to explain the success of social insect communication. These pheromones may be secreted in dejection as well which allows tracing and returning to the nest. Nevertheless, little is known about the role and function of these pheromones in bed bugs.

***Recruitment pheromones*** induce nestmates to leave the nest. They are not, however, restricted to the social insects and are found in a variety of taxa, although it has been understudied in bed bugs [88].

***Royal pheromones*** used by queens in social insects enable workers to recognize and care for these vital individuals [89]. Bed bugs are gregarious but are not strictly social insects, and there are no castes in bed bug colonies. However, the role of this pheromone has been understudied in cimicidae bugs.

In spite of the investigations carried out on VOCs and their impacts on bed bugs, the role and impact of some of the aforementioned pheromones on these ectoparasites are largely unknown. Therefore, there is a serious need for further in-depth investigations with more focus on the role of the mentioned pheromones so that in the following steps they can be used for bed bug detection and control measures.

### 1.3. Target-Specific Role of VOCs

The insects of cimicidae (Hemiptera) family are well known for their unpleasant characteristic odor, which is caused by the release of aldehydes and esters by these bugs. The types of molecules that these insects release against various stimuli are different. These compounds are stored in three pairs of dorsal abdominal glands (DAGs) in the nymphs, whereas they are produced in the metathoracic glands (MTGs) and stored in an orange-colored reservoir between these glands in the adults [90]. Several studies in the literature have been reported the chemical composition of the compounds stored in these glands, but only a few studies have considered the role of these compounds. Each of mentioned molecules has a target-specific role in life cycle of bed bugs. Detailed information on the VOCs and their role so far identified in the bed bugs is given in Table 1.

### 1.4. VOC-Based Sampling and Analyzing Methods

Sampling and analyzing of bed bug VOCs are rather difficult tasks, largely due to the low quantities released and rapid dispersion into the air. The development of techniques that can rapidly detect these volatile compounds in bed bugs is therefore of great interest. Regarding the presence of VOCs in a gaseous state, the gas-based sampling and detecting methods are valuable as they operate in the gas phase. There are two sampling methods of VOCs:

“Active sampling” is the most common sampling procedure in which the VOCs are collected on an adsorbent tube using an air sampling pump. All released semiochemicals are deposited onto sorbents (e.g., porous polymers, active fibers or coated materials), and, once collected, they are subsequently desorbed using organic solvents or thermal protocols. These techniques consist of the confinement of a portion of VOC-containing atmosphere inside a recipient, e.g., canister, cuvette, flask or a special bag. The VOC collection can, therefore, be performed by means of a pump or ventilator, trapping them in a sorbent material. However, diverse factors should be considered for avoiding the loss of some compounds, such as temperature, relative humidity, light exposure, containing recipient surface and sorbent materials or the control of reactive species (e.g., oxidizers), therefore to ensure reliable measurements [68]. “Passive Sampling” is another sampling method based on controlled diffusion of vapors from ambient air into the adsorbent pad of the Diffusive Sampler (called a Diffusive Badge). However, it is less accurate than active sampling. The collected VOCs are subsequently analyzed through analytical platforms. There are a wide range of gas analytic technologies of VOCs released by bed bugs. Gas chromatography–mass spectrometry (GC-MS) is a common analytical method used for VOCs detection [92,93]. However, this reference method of detection is expensive and time-consuming and thus requires training and expertise. Consequently, inexpensive, portable and user-friendly methods are required. A list of sampling methods used for identification of bed bugs’ VOCs is given in Table 2.

The detection and measurement of bed bug VOCs in an infested area are commonly performed by portable air samplers possessing absorbing tubes, e.g., Tenax (for VOCs and SVOCs). The VOCs are adsorbed through the mentioned tubes and afterward desorbed either thermally (Tenax) or by elution (DNPH) and then analyzed by GC-MS/FID or HPLC [94]. Currently, gas chromatography coupled with a high-resolution detection mode (e.g., flame ionization (FID), electron capture (ECD), photoionization (PID) or mass spectrometry (MS)) is commonly used for detection, identification and quantification of bed bug VOCs [90].

### 1.5. VOC Applications

Regarding toxicity and increasing cases of bed bug resistance against chemical insecticides, the integrated pest management (IPM) systems recently operate more by limiting applications of chemical insecticides and placing more focus on the use of naturally occurring control methods [92,95,96]. With infestations being difficult to identify and eliminate in the early stages, there is a need for a monitoring tool that could be used for surveillance, evaluation of intervention success and even mass trapping. Tremendous progress has been made in understanding insect olfaction mechanisms, leading to increased interest in how insects are affected behaviorally by VOCs and raising opportunities for applying this knowledge in integrated pest management (IPM) strategies [97].

Bed bugs, under specific conditions such as stress, emit specific odors. These odors are comprised of VOCs which, depending on their volatility, persist in a gaseous state. The exuviae and excretion of dead and living bed bugs together possess diverse VOC profiles, providing a foundation for successful detection of bed bug infestations. The most common application of VOCs in bed bug management is often based on the use of VOCs guiding the bed bugs during their search for blood-feeding or mating [19,98]. Bed bug aggregation pheromones are also considered a promising attractant for use in the monitoring, treatment efficacy evaluation, mass trapping efforts and management of bed bugs [99]. Adhesive or pitfall traps coupled with these attractive VOCs are passive monitoring techniques that can greatly contribute to reducing bed bug infestations [19,94,100]. Active monitors are equipped with heat and/or chemical attractants that draw the bed bugs and therefore increase the success of detection [19]. The combination of a sugar–yeast monitor with a chemical lure (e.g., nonanal, L-lactic acid, 1-octen-3-ol and spearmint oil) is an affordable and effective tool for monitoring bed bugs [101]. This monitor is especially useful for monitoring bed bugs where a human host is not present.

In addition to attractive VOCs, the traps containing large amounts of some sexual or aggregation VOCs are placed in the field practices to confuse bed bug males and females, making it difficult for them to find each other to mate [89].

On the other side, chemical repellents as alternatives to insecticides are now playing a significant role in pest control [102,103]. Some terpene-derived chemicals that are both effective and eco-friendly for insect control have been used extensively to interrupt the host-seeking process of the blood-feeding arthropods [104,105]. Harraca et al. [85] tested the olfactory responses of *C. lectularius* to nearly 30 chemicals including five chemical repellents. Liu et al. [106] conducted a systematic study characterizing the electrophysiological responses of olfactory sensillum in the common bed bug to 52 chemicals reported as repellents for different insects.

## 2. Materials and Methods

**Figure 3:**
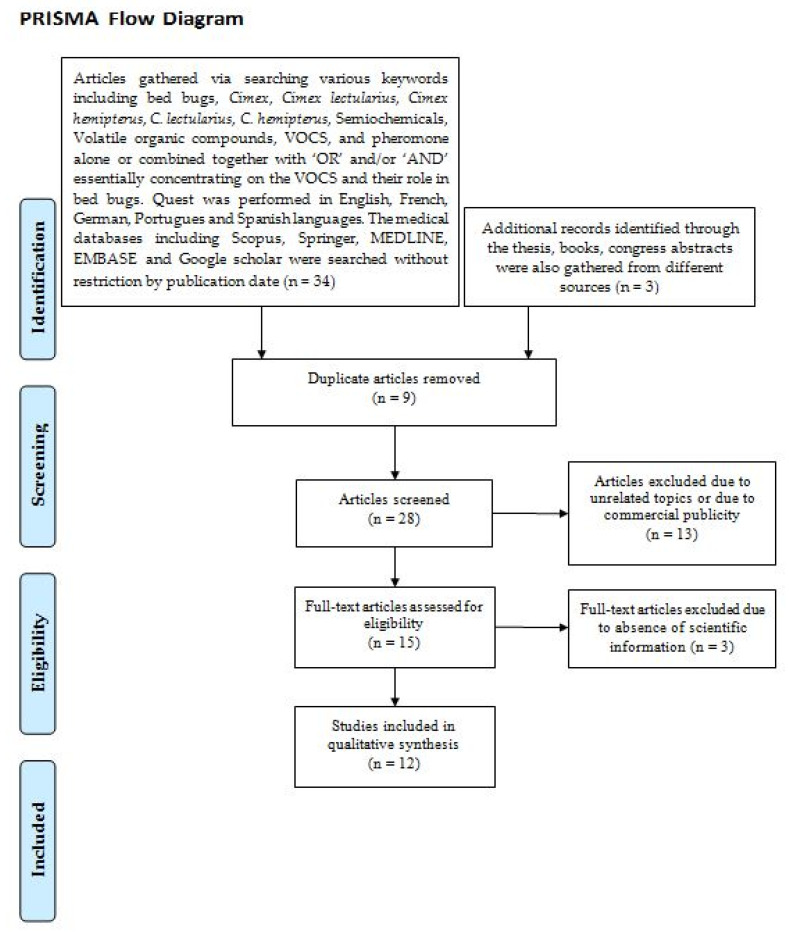
The literature quest strategy used in the present study.

To explore the detailed characteristics of the VOCs detected in bed bugs, a narrative review was performed on the released literature, including research articles, books and dissertations according to the PRISMA (Preferred Reporting Items for Systematic Reviews and Meta-Analyses) guideline [107]. The searches were performed in Scopus, PubMed, Science Direct, ProQuest, Web of Science, Springer, MEDLINE and Google Scholar in five languages (English, French, German, Portuguese and Spanish) without restriction by publication date. The relevant articles that met the mentioned criteria were selected. Duplicated articles and those with unrelated topics were excluded. A total of 34 articles published on the mentioned subjects were gathered. Among them, 12 articles that met the study criteria were selected (Figure 3). Detailed information of the investigations performed on the VOCs in the released literature is given in Table 3.

## 3. Discussion

An overview of the released literature on the VOCs in bed bugs allowed us to explore 12 studies conducted on *C. lectularius* and *C. hemipterus* as human ectoparasites. Most of the investigations were carried out on *C. lectularius* (10 studies) compared to two on *C. hemipterus*. Laboratory strains (eight studies) were the most frequent specimens that underwent detection for VOCs.

So far, 49 volatile molecules have been detected in bed bugs. They included 17 aldehydes, seven ketones, five alcohols, six acids, four esters, four terpenes, two organic sulfure compounds, two hydrocarbons, one Amid and one Alkane. Of the 49 detected molecules, 26 molecules were identified in *C. hemipterus*, and the remaining were identified in *C. lectularius*.

Concerning *C. lectularius*, aldehydes (13 molecules), ketones (six molecules) and terpens (six molecules) were the most frequent components of analyzed VOCs, while for *C. hemipterous*, aldehydes (10 molecules) and acids (six molecules) were the most abundant substances reported in the processed specimens. To the best of our knowledge, no acid or hydrocarbon were reported in *C. lectularius*. Similarly, ester and terpene molecules were not detected in *C. hemipterus*. Aldehydes were the common molecules detected in both *C. lectularius* and *C. hemipterous* specimens with (E)-2-hexenal and (E)-2-octenal substances being the most abundant ones (ratio 3:1 in adults and 3:7 in 4th and 5th instar nymphs) [24] (Table 1 and Table 3).

Based on earlier studies on the VOCs in bed bugs, females emit approximately equal amounts of (E)-2-hexenal and (E)-2-octenal, whereas males emit much more (E)-2-hexenal rather than (E)-2-octenal [76], while according to another investigation, there was no significant difference in the amount of (E)-2-hexenal and (E)-2-octenal emitted by female and male specimens [64]. In addition, it was shown that the males release approximately five times more (E)-2-hexenal and (E)-2-octenal than females and at least 50 times more than juveniles [24]. Furthermore, four aldehydes of (E)-2-hexenal, 4-oxo-(E)-2-hexenal, (E)-2-octenal and 4-oxo-(E)-2-octenal were consistently detected in bed bugs’ exuviae regardless of the instars from which the exuviae were obtained [38]. Conversely, dead bed bugs and exuviae had the lowest VOC profile which indicates a key distinction between the VOCs released from living and dead bed bugs [38].

Regarding the role of VOCs, they are commonly characterized using the olfactometry system. Olfactometers are used to gauge the VOC detection threshold of substances in a precise and controlled manner. To measure intensity, olfactometers introduce one or multiple VOCs as a baseline to find the examined specimens’ behaviors [109]. The life stage of bed bugs, their VOC concentrations, the distance between a VOC source and bed bugs and the temperature are important factors affecting olfactometric assessment [55,84,110,111]. Herein, we provided a detailed list of VOCs detected in both sexes of bed bugs (Table 1). Of the 49 detected VOCs, molecules with an aggregation role (46 molecules) were the most reported ones in processed bed bugs followed by sexual (11) and alarm (4) substances. Some reported VOC roles in the examined specimens were controversial because for the same molecule(s), diverse roles were reported simultaneously. For instance, (E)-2-hexenal and (E)-2-octenal were identified as essential components of the bed bugs’ aggregation pheromones [84]. Other than their aggregation role, it is actually clear that these volatiles have additional functions as defensive chemicals that are released in high concentrations [1,54,112,113]. The latter commonly occurs when bed bugs are attacked by predators (e.g., bats or ants) [54], are encountered with high concentrations of carbon dioxide [76] or are encountered with undesirable mating activities [84,91]. It seems that the function type (aggregation versus defense) of these VOCs largely depends on the released concentration. Therefore, (E)-2-hexenal and (E)-2-octenal serve as multifunctional pheromone components that are attractive/arrestant at low concentrations but repellent at high concentrations [109]. Acetaldehyde (C_2_H_4_O), (E)-2-hexenal (C_6_H_10_O) and Butan-2-one (C_4_H_8_O) were also reported with aggregation and alarm roles [60,70,72]. Furthermore, Octanal (C_8_H_16_O) and Geranyl acetone (C_12_H_22_O) were detected as aggregation and sexual VOCs [22,99]. Although there is no description explaining these incompatibilities in reported roles, they may be linked to the bed bug species analyzed (*C. lectularius* versus *C. hemipterus*), the VOC concentrations detected, lifecycle (egg, larva, nymph and adult), sex (male versus female) or the analyzing condition of processed bed bugs.

VOC processing methods/devices are another essential element in bed bug detection. According to the literature, various methodologies were used for VOC detection in bed bugs. Among them, GC-MS was the most frequent analyzing method used (Table 3). Depending on the type of trapping method (absorbent) and device, they were used for detection, identification and quantification of various VOCs. Despite the great advantages of the aforementioned methods, they have some drawbacks which limit their application in the field, such as being bulky, expensive and needing expertise. Therefore, developing sensitive hand-held portable devices involving gas chromatography analyzers that do not have the mentioned limitations is a fundamental step in the on-site detection of bed bugs in infested locations. This has the advantages of rapidity, simplicity and avoiding multiple inspections. Regarding the lack of need for resident rehousing or moving due to the detection of bed bug VOCs which are performed by air sampling, this practice represents an attractive method from the users’ point of view. Furthermore, re-inspection of the previously infested location by VOC analysis to continue or terminate the treatment is rather rapid, which therefore makes this detecting intervention economical. This promising approach can be used in industries, hotels, hospitals, touristic centers, etc., for quick diagnosis of probable infestation and subsequent control management strategies against bed bugs.

## 4. Conclusions

The communication between bed bugs themselves and with their environment is mediated by chemical interactions especially through VOCs released by an individual and received by another one. Such chemical-based communication is intimately involved with various behaviors of bed bugs, such as defense, mating or aggregation. Bed bugs emit various VOCs with different concentrations depending on their sex and life stages. While the number of bed bug specimens may have an effect on the amount of VOCs available for detection, developing the analyzing methods which are able to detect, identify and quantify the VOCs is of great importance for bed bug management. A literature review of the investigations carried out on VOC semiochemicals allowed us to explore 12 studies conducted on *C. lectularius* (10 studies) and *C. hemipterus* (2) mostly performed on laboratory strains (8 studies). We also highlight the identification of 49 VOCs in *C. lectularius* (23 molecules) and *C. hemipterus* (26) which are emitted by both sexes during diverse compartments including aggregation (46), mating (11), defense (4), etc., and all life stages including exuviae or bed bug death as principal indicators of infestation. The aldehydes (17 molecules) were the common molecules detected in both *C. lectularius* and *C. hemipterous* specimens with (E)-2-hexenal and (E)-2-octenal substances as the most abundant ones. They were detected and analyzed by various methodologies. Among them, GC-MS was the most frequent analyzing method used. This identification of VOCs responsible for chemical communication of bed bugs allows for their further application in control of bed bugs singly or in integration with other control strategies and can be involved in control management strategies against these ectoparasites in private (e.g., individual or collective dwellings) or public (e.g., hotels, hospitals and transportation) settings and other human dwellings.

## Figures and Tables

**Table 1:** Volatile organic compounds detected in the bed bugs and their known functions.

VOCs		Molecular Formula	Chemical Group	Bed Bug Species	Behavioral Role	References
1	Acetaldehyde	C_2_H_4_O	Aldehyde	*C. hemipterus*	Aggregation	[72]
				*C. lectularius*	Alarm	[62]
2	Benzaldehyde	C_7_H_6_O	Aldehyde	*C. lectularius*	Aggregation	[24]
				*C. hemipterus*	Aggregation	[72]
3	Butanal	C_4_H_8_O	Aldehyde	*C. lectularius*	Aggregation	[65]
4	(E)-2-hexenal	C_6_H_10_O	Aldehyde	*C. hemipterus*	Aggregation	[72]
				*C. lectularius*	Alarm	[62,65]
5	Heptanal	C_7_H_14_O	Aldehyde	*C. lectularius*	Sexual *	[91]
6	Hexanal	C_6_H_12_O	Aldehyde	*C. hemipterus*	Aggregation	[72]
7	Octanal	C_8_H_16_O	Aldehyde	*C. lectularius*	Aggregation	[24]
				*C. lectularius*	Sexual *	[91]
8	Pentanal	C_5_H_10_O	Aldehyde	*C. lectularius*	Aggregation	[65]
9	Propanal	C_4_H_8_O	Aldehyde	*C. lectularius*	Sexual *	[91]
				*C. hemipterus*	Aggregation	[72]
10	Nonanal	C_9_H_18_O	Aldehyde	*C. lectularius*	Aggregation	[24]
				*C. lectularius*	Sexual *	[91]
11	Undecanal	C_11_H_22_O	Aldehyde	*C. lectularius*	Sexual *	[91]
12	(E)-heptenal	C_7_H_12_O	Aldehyde	*C. hemipterus*	Aggregation	[72]
13	(E)-2-octenal	C_8_H_14_O	Aldehyde	*C. lectularius*	Aggregation	[24,49,65,70,91]
				*C. hemipterus*	Aggregation	[72,76]
14	(E,Z)-2,4-Octadienal	C_8_H_12_O	Aldehyde	*C. lectularius*	Aggregation	[24]
15	3-methylthio-propanal	C_4_H_8_OS	Aldehyde	*C. hemipterus*	Aggregation	[72]
16	4-oxo-(E)-2-octenal	C_8_H_12_O_2_	Aldehyde	*C. lectularius*	Alarm	[76]
17	4-oxo-(E)-2-hexenal	C_6_H_8_O_2_	Aldehyde	*C. hemipterus*	Aggregation	[72]
18	Acetophenone	C_8_H_8_O	Ketone	*C. lectularius*	Aggregation	[65]
19	Acetone	C3H6O	Ketone	*C. lectularius*	Sexual *	[91]
20	Butan-2-one	C_4_H_8_O	Ketone	*C. lectularius*	Aggregation	[62]
				*C. hemipterus*	Alarm	[72]
21	Geranyl acetone	C_13_H_22_O	Ketone	*C. lectularius*	Aggregation	[24]
					Sexual *	[91]
22	Sulcatone (6-Methyl-5-hepten-2-one)	C_8_H_14_O	Ketone	*C. lectularius*	Aggregation	[24]
23	2-octanone	C_8_H_16_O	Ketone	*C. hemipterus*	Aggregation	[72]
24	2-hexanone	C_6_H_12_O	Ketone	*C. lectularius*	Aggregation	[65]
25	Acetamide	C_2_H_5_NO	Acid	*C. hemipterus*	Aggregation	[72]
26	Hexanoic acid	C_6_H_12_O_2_	Acid	*C. hemipterus*	Aggregation	[72]
27	Phenyl acetic acid	C_8_H_8_O_2_	Acid	*C. hemipterus*	Aggregation	[72]
28	2-methyl propanoic acid	C_8_H_16_O_3_	Acid	*C. hemipterus*	Aggregation	[72]
29	(E)-2-hexenoic acid	C_6_H_10_O_2_	Acid	*C. hemipterus*	Aggregation	[72]
30	(E)-2-octenoic acid	C_8_H_14_O_2_	Acid	*C. hemipterus*	Aggregation	[72]
31	Methyl nonanoate	C_10_H_20_O_2_	Ester	*C. lectularius*	Aggregation	[65]
32	Ethyl octanoate	C10H20O2	Ester	*C. lectularius*	Aggregation	[65]
33	Pentyl hexanoate	C11H22O2	Ester	*C. lectularius*	Aggregation	[65]
34	Benzyl Acetate	C_6_H_5_CH_2_OCOCH_3_	Ester	*C. lectularius*	Aggregation	[24]
35	(+) Limonène	C_10_H_16_	Terpene	*C. lectularius*	Aggregation	[24]
					Sexual *	[91]
36	(-) Limonène	C_10_H_16_	Terpene	*C. lectularius*	Aggregation	[24]
				*C. lectularius*	Sexual *	[91]
37	Verbenone	C_10_H_14_O	Terpene	*C. lectularius*	Aggregation	[65]
38	Decanal	C_10_H_20_O	Terpene	*C. lectularius*	Aggregation	[24]
				*C. lectularius*	Sexual *	[91]
39	Benzyl alcohol	C_6_H_5_CH_2_OH	Alcohol	*C. lectularius*	Aggregation	[24,65]
					Sexual *	[91]
40	Diethylene glycol	C_4_H_10_O_3_	Alcohol	*C. hemipterus*	Aggregation	[72]
41	2-ethyl-1-hexanol	C_8_H_18_O	Alcohol	*C. hemipterus*	Aggregation	[72]
42	2-propyl-1-pentanol	C_8_H_18_O	Alcohol	*C. hemipterus*	Aggregation	[72]
43	2-isopropyl-5-methyl-cyclohexanone	C_10_H_16_O_2_	Alcohol	*C. hemipterus*	Aggregation	[72]
44	Tetradecane	C_14_H_3_0	Hydrocarbon	*C. hemipterus*	Aggregation	[72]
45	Azulene	C_10_H_8_	Hydrocarbon	*C. hemipterus*	Aggregation	[72]
46	Pyrrolidin-2-one	C_4_H_7_NO	Amid	*C. hemipterus*	Aggregation	[72]
47	Tridecane	C_13_H_28_	Alkane	*C. hemipterus*	Aggregation	[72]
48	Dimethyl disulfide	C_2_H_6_S_2_	Organic Sulfur Compound	*C. lectularius*	Aggregation	[65]
49	Dimethyl trisulfide	C_2_H_6_S_3_	Organic Sulfur Compound	*C. lectularius*	Aggregation	[65]

*: VOCs detected during sexual activity.

**Table 2:** Various trapping methods used for bed bugs’ VOCs detection.

Trapping Technology	Adsorbent Polymer	References
SPME (Solid Phase Micro Extraction)	DVB/CAR/PDMS	[25,70]
NTD (Needle Trap Device)	HaySep Q divinyl benzene	[25]
TFME (Thin Film Microextraction)	PDMS	[25]
Active adsorbent sampling	Poropak Q trap elution	[24]
Active adsorbent sampling	TENAX TA/Carbograph 5D *	[91]
SPME	Carboxen/PDMS	[49]
Active adsorbent sampling	TENAX GR	[65]
SPME	Carboxen/PDMS	[70]
Liquid extraction (water/ethanol)	NA	[62]
Active adsorbent sampling	TENAX GR	[76]
Methanol extraction	NA	[72]
SPME	Carbon WR/PDMS	[26]

*: PTR-MS; NA: Not available.

**Table 3:** Detailed profiles of investigations carried out on the bed bugs’ volatile organic compounds in the literature.

Author(s)	Entomological Criteria					Analysing Method	Molecules Identified
	Species	Life Stage	Sex	Fed/Unfed	Field/Laboratory		
Levinson et al. [62]	*C. lectularius*	Larva & adult	♂ & ♀	Fed	NA	GC-MS	Acetaldehyde; Butan-2-one; (E)-2-hexenal; Sulcatone (6-Methyl-5-hepten-2-one)
Siljander et al. [24]	*C. lectularius*	Larva & adult	♂ & ♀	Fed	Laboratory	GC-MS	(E)-2-hexenal; Benzaldehyde; Benzyl alcohol; (E,Z)-2,4-Octadienal; Sulcatone (6-Methyl-5-hepten-2-one); Octanal; Limonène; Nonanal; Benzyl Acetate; Decanal; Geranyl acetone ((E)-6,10-Dimethyl-5,9-undecadien-2-one)
Liedtke et al. [76]	*C. hemipterus*	Nymph & adult	♂ & ♀	Fed	Laboratory	GC-MS	(E)-2-hexenal; 4-oxo-(E)-2- hexenal; (E)-2-octenal; 4-oxo-(E)-2-octenal
Kilpinen et al. [91]	*C. lectularius*	Adult	♂ & ♀	Fed	Laboratory	GC-MS	Acetone; Propanal; (E)-2-hexenal; Hexanal; Benzaldehyde; Benzyl alcohol; Heptanal; (E)-2-octenal; Sulcatone (6-Methyl-5-hepten-2-one); Octanal; Limonène; Nonanal; Decanal; Undecanal; Geranyl acetone ((E)-6,10-Dimethyl-5,9-undecadien-2-one)
Eom et al. [25]	*C. lectularius*	All stages	♂ & ♀	NA	Field	GC-MS	Phenyl-1,3,3-trimethylindan; Heptadecane; 2,6,10,14-Tetramethylpentadecane; Hexyl cinnamic aldehyde; Octadecane; Isopropyl myristate; Galaxolide; 7-Methyl-Z-tetradecen-1-ol acetate; 2-Methylhexadecan-1-ol; Methyl hexadecanoate; Dibutyl phthalate; Ethyl hexadecanoate; Isopropyl palmitate; 8-Octadecenal; Methyl 4-hydroxyoctadecanoate; Z-5-methyl-6-heneicosen-11-one
Mendki et al. [72]	*C. hemipterus*	Nymph	-	NA	Laboratory	GC-MS	Acetaldehyde; Acetamide; Pyrrolidin-2-one; 2-methyl propanoic acid; (E)-2-hexenal; Hexanal (E)-2-hexenol; 3-methylthio-propanal; Benzaldehyde; Diethylene glycol;(E)-heptenal; (E)-2-hexenoic acid; Hexanoic acid; (E)-2-octenal; Dimethyl trisulfide; Azulene; 2-octanone; 2-ethyl-1-hexanol; 2-propyl-1-pentanol; Phenyl acetic acid; (E)-2-octenoic acid; 2-isopropyl-5-methyl-cyclohexanone (menthone); Tridecane; Tetradecane
Gries et al. [65]	*C. lectularius*	Egg, nymph, adult & exuviae	♂ & ♀	Fed & ufed	NA	GC-MS	Butanal; Pentanal; Dimethyl disulfide; (E)-2-hexenal; Hexanal; 2-hexanone; Benzaldehyde; Benzyl alcohol; Acetophenone; (E)-2-octenal; Dimethyl trisulfide; Verbenone; Methyl nonanoate; Ethyl octanoate; Pentyl hexanoate
Choe et al. [49]	*C. lectularius*	Nymph & adult	♂ & ♀	Fed	Laboratory	GC-MS	(E)-2-hexenal; 4-oxo-(E)-2- hexenal; (E)-2-octenal; 4-oxo-(E)-2-octenal
Olson et al. [70]	*C. lectularius*	Adult	♂ & ♀	Fed	Field	GC-MS	(E)-2-hexenal; (E)-2-octenal
Zhang et al. [108]	*C. lectularius*	Egg, nymph & adult	♂ & ♀	Fed	Laboratory	GC-MS	(E)-2-hexénal; (E)-2-octénal (adult); Eucalyptol (egg)
Weeks et al. [99]	*C. lectularius*	Larva & adult	♂ & ♀	Fed	Laboratory	GC-EAG	Hexanal; Heptanal; Benzaldehyde; (RS)-1-Octen-3-ol; Octanal; 3-Carene; β-Phellandrene; (E)-2-Octenal; (3E,5E)-Octadien-2-one; Nonanal; (E)-2-Nonenal; 2-Decanone; Decanal; Dodecane; Nonanoic acid; 2-(2-Butoxyethoxy) ethyl acetate; (E)-2-Undecenal; (S)-Germacrene D
Cannon et al. [26]	*C. lectularius*	Egg & adult	NA	Fed	Laboratory	GC-MS	Acetone; (2-aziridinylethyl) amine; toluene; octane; hexanal; N,N-dimethylformamide; ethylbenzene; m-xylene; 2-héxanal; p-xylene; heptanal; α-pinene; 2-butoxyethanol; 4-ethyloctane; 5-methylnonane,2,2,6-trimethyloctane; 2-trifluoroacetoxydodecane; decane; 2-tridecyl ester methoxyacetic acid; α-methylstyrene; benzaldehyde; 2,2-dimethyldecane; 2,2,4,6-6-pentamethylheptane; 3-8-dimethylundecane, α-methyl-α-[4methylpentyl]oxiranmethanol; 1-(2-methoxy-1-methylethoxy)-2-propanol; 2,2-dimethyl-1-octanol; 2,7,10-trimethyldodecane; 5-ethyl-2,2,3-trimethylheptane; 3,6-dimethylundecane; 2,6,8-trimethyldecane; 2-ethyl-1-hexanol; 2,3,4-trimethyldecane; 2,2,7,7-tetramethyloctane; 3,7-dimethyldecane; 4-methylundecane; undecane 2-octenal; 2-hexyl-1-octanol; 6-methyloctadecane; 4-ethyl-2,2,26,6-tetramethylheptane; N-[5-(2-hydroxyphenyl)-1,3,4-thiadiazol-2-yl]benzamide; nonanal; 5-methylundecane; 2,6,10-trimethyldodecane; 3-methylundecane; 9-methylheptadecane; (E)-2-dodecene; dodecane; Z,Z-2,5-pentadecadien-1-ol; 2-methyl-1-hexadecanol; 1-methyl-4-(1-methylethyl)-cyclohexanol; tridecane; 2-azido-2,4,4,6,6,8,8-heptamethylnonane; tetradecane; 3-Hydroxy-2,2,4-trimethylpentyl 2-methylpropanoate; 3-(isobutyryloxy)-1-isopropyl-2,2-dimethylpropyl-2-methylpropanoate

GC-MS: Gas chromatography-Mass spectrometry; SPME: Solid phase micro extraction; GC-EAG: Gas chromatography-electroantennography; NA: Not available.

## Data Availability

Not applicable.
